# An Efficient Dynamic Solution for the Detection and Prevention of Black Hole Attack in VANETs

**DOI:** 10.3390/s22051897

**Published:** 2022-02-28

**Authors:** Abdul Malik, Muhammad Zahid Khan, Mohammad Faisal, Faheem Khan, Jung-Taek Seo

**Affiliations:** 1 Network System & Security Research Group, Department of Computer Science & IT, University of Malakand, Chakdara 18800, Pakistan; qariabdulmalik@uom.edu.pk (A.M.); mzahidkhan@uom.edu.pk (M.Z.K.); mfaisal@uom.edu.pk (M.F.); 2 Department of Computer Engineering, Gachon University, Seongnam 13120, Korea

**Keywords:** AODV, BHA, IoT, network security, VANET

## Abstract

Rapid and tremendous advances in wireless technology, miniaturization, and Internet of things (IoT) technology have brought significant development to vehicular ad hoc networks (VANETs). VANETs and IoT together play a vital role in the current intelligent transport system (ITS). However, a VANET is highly vulnerable to various security attacks due to its highly dynamic, decentralized, open-access medium, and protocol-design-related concerns. Regarding security concerns, a black hole attack (BHA) is one such threat in which the control or data packets are dropped by the malicious vehicle, converting a safe path/link into a compromised one. Dropping data packets has a severe impact on a VANET’s performance and security and may cause road fatalities, accidents, and traffic jams. In this study, a novel solution called detection and prevention of a BHA (DPBHA) is proposed to secure and improve the overall security and performance of the VANETs by detecting BHA at an early stage of the route discovery process. The proposed solution is based on calculating a dynamic threshold value and generating a forged route request (RREQ) packet. The solution is implemented and evaluated in the NS-2 simulator and its performance and efficacy are compared with the benchmark schemes. The results showed that the proposed DPBHA outperformed the benchmark schemes in terms of increasing the packet delivery ratio (PDR) by 3.0%, increasing throughput by 6.15%, reducing the routing overhead by 3.69%, decreasing the end-to-end delay by 6.13%, and achieving a maximum detection rate of 94.66%.

## 1. Introduction

A vehicular ad hoc network (VANET) is a special type of mobile ad hoc network (MANET) in which vehicles and roadside units (RSUs) are linked to create a safer and more efficient driving environment [1]. A typical VANET architecture consists of three primary components, namely, onboard units (OBUs), roadside units (RSUs), and trusted authority (TA) [1,2,3]. Every vehicle has an OBU that collects, analyses, and transmits information to other vehicles in the vicinity. An RSU is installed along the roadside that is used to communicate with vehicles, infrastructure, and a TA. In essence, a TA is a registration unit that manages the VANET system by registering the OBUs, RSUs, and vehicle users. A VANET is the backbone of the intelligent transportation system (ITS) and it plays a crucial role in supplying real-time and sensitive information to the drivers and traffic authorities [4,5]. Another key component of an ITS is the IoT [6], which transforms conventional VANETs into the Internet of vehicles (IoV), enabling data collection and sharing data about infrastructures, vehicles, humans, and road conditions [7,8,9].

**Figure 1:**
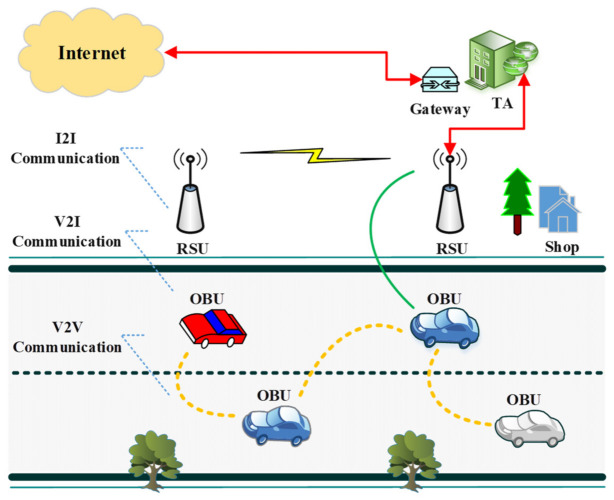
Generic architecture of a VANET.

The primary distinction between a MANET and a VANET is their MAC addressing, as a MANET operates on IEEE 802.11m and a VANET operates on IEEE 802.11p technology [10]. In a MANET, the movement of nodes is random, while in a VANET, some nodes are fixed (RSUs) and others (vehicles) travel at high speed along the roadside. A VANET’s nodes have unlimited energy and processing power, whereas a MANET lacks these features [11]. Within a VANET, communications are divided into three distinct categories: vehicle-to-vehicle (V2V), vehicle-to-infrastructure (V2I), and infrastructure-to-infrastructure (I2I) communications [12,13]. V2V communication is purely on an ad hoc basis, which allows for the exchange of information between vehicles over a short range. V2I communication provides information to vehicles and static infrastructures. Meanwhile, I2I communication provides additional traffic information over 3G/4G channels, which is important for driver assistance and vehicle tracking. The generic architecture of a VANET is shown in Figure 1.

The high mobility of vehicles, high dynamic network topology, non-centralized control, large scale network, time-critical communications, and open access to both legitimate and illegitimate users are some of the distinguishing characteristics of VANETs [14,15,16,17]. In VANETs, data communication and routing are constantly vulnerable to many security attacks due to these characteristics and constraints. As a result, one of the primary considerations in VANET applications is to secure communications. However, in VANETs, data transmission between two nodes requires the assistance of intermediate nodes to transfer the data because the destination node is not often lying in the transmission range of the source, hence routing protocols are used to establish the best route between nodes. Over time, various routing protocols and security mechanisms have been developed [18]. Out of these, the ad hoc on-demand distance vector (AODV) [19] was found to be one of the most famous and commonly used routing protocols in VANETs [20,21]. AODV is also known as a demand-driven protocol since it discovers a new route only when it is required, rather than in advance. AODV provides a fast, dynamic network connection with little processing overhead and memory requirements, making it an ideal choice for a highly dynamic VANET [22,23]. However, there are several significant security vulnerabilities and challenges with the AODV protocol that must be addressed. For instance, the source node is always unaware of the intended destination. Such features of AODV make VANETs more vulnerable to various security attacks, such as a wormhole attack, black hole attack (BHA), and gray hole attack (GHA) [2,16,24,25].

Secure and efficient communications in VANETs are very essential because the vehicles are moving quickly, and the information is often safety related and time sensitive. Ensuring the security of the messages generated by the vehicles is very crucial, as the nodes in VANETs exchange them in the open wireless medium. Due to the presence of the aforementioned attacks, the applications and services of VANETs are compromised. One such kind of attack is a BHA in which a malicious node completely drops the packets instead of forwarding them onto its final destination. These packets may contain important emergency messages and warning alerts. A BHA drops such packets, which results in degradation of the overall network security, performance, and disruption in the network information-sharing process. Road accidents are a significant cause of deaths and physical disabilities. Hence, dropping all such packets in a highly dynamic VANET could result in road fatalities, accidents, traffic jams, and congestions. Motivated by this, in this study, we proposed a novel and efficient solution for the detection and prevention of a well-known security attack BHA in the AODV routing protocol to improve the overall security and performance of VANETs. The solution was based on calculating a dynamic threshold value from sequence numbers of RREPs and generating a forged RREQ packet. In a nutshell, the proposed solution increased the PDR and network throughput while reducing the routing overhead and end-to-end delay.

The rest of the manuscript is organized as follows: Section 2 provides a brief background of BHAs in VANETs, Section 3 describes the related work, Section 4 explains the proposed work, Section 5 discusses the implementation and evaluation, and Section 6 concludes and gives future direction to the research work.

## 2. Black Hole Attacks (BHAs) in VANETs

The highly dynamic, open-access medium, distributed infrastructure, and protocol designing issues have made VANETs vulnerable to many security attacks, such as a denial of service (DoS) attack, Sybil attack, wormhole attack, flooding attack, impersonation attack, jellyfish attack, GHA, and BHA [2,16,24,25]. Due to the presence of these attacks, the applications and services of VANETs can be compromised.

A BHA is a type of DoS in which a malicious node completely drops packets from the legitimate node. In a BHA, when a malicious node receives an RREQ packet from the source node, it quickly responds with a fake RREP without checking its routing table. This RREP packet contains a higher sequence number and minimum hop count value, which is considered to be the freshest and shortest route in AODV [26,27]. Once the source node receives the fake RREP packet, it deceptively considers it an optimized path and starts transferring data packets toward the black hole node. A BHA drops such packets instead of forwarding them to their final destination, which results in degradation of the overall network security and performance, as well as disruption in the network information-sharing process. These packets may contain critical information messages, such as emergency notifications and warning alters, which must be delivered quickly and within a specific time frame. Dropping such packets in a highly dynamic VANET could result in road fatalities, accidents, traffic jams, and congestion. Our research focus in this study was to address the BHA issue in VANETs and propose a new, more efficient solution. Because a BHA is one of the most serious attacks in VANETs, it serves as the foundation for DoS attacks in which the network service is unavailable to the intended users.

**Figure 2:**
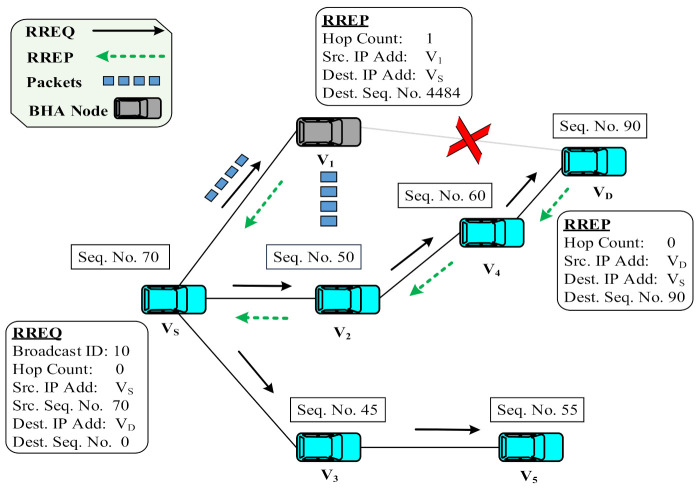
Black hole attack.

In the above Figure 2, a BHA in the AODV protocol is explained with the help of an example scenario. For instance, source vehicle vs. wants to communicate with destination vehicle V_D_. vs. broadcasts an RREQ packet to all its neighboring vehicles, i.e., V_1_, V_2_, and V_3_. Upon receiving the RREQ, V_1_ quickly responds with a fake RREP containing a spoof higher destination sequence number (DSN) value (4484). Meanwhile, vehicles V_2_ and V_3_ increase their hop count values by one in the RREQ packet and broadcast it further to their next-hop vehicles. In the meantime, vs. receives the first RREP from V_1_. Therefore, source vehicle vs. selects a route to destination V_D_ that goes through V_1_ (i.e., black hole attacker) and starts transferring data packets. Upon receiving the packets, V_1_ drops all these packets rather than forwarding them to V_D_. The RREP(s) that arrives later is discarded by the source vehicle V_S_.

**Figure 3:**
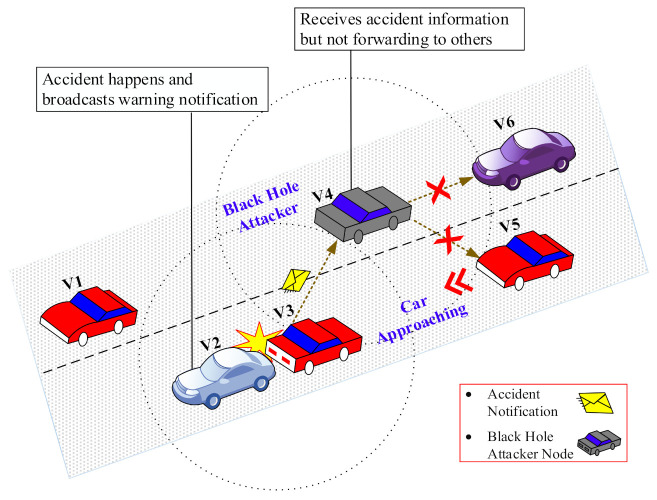
A visual representation of the impact of a BHA on VANET.

Figure 3 shows a visual representation of the impact of BHA on VANET. In this figure, a collision occurs between two vehicles and a warning alert is sent by vehicle V_3_ to vehicle V_4_ (BHA vehicle). V_4_ drops the warning alert instead of forwarding it to the approaching vehicles, i.e., V_5_ and V_6_. As a result, it could lead to more accidents, hazards, and traffic jams.

## 3. Related Work

Concerning the mitigation of BHA and eradication of malicious nodes in VANETs, over time, many solutions were proposed and reported in the relevant literature. One such notable related work was proposed by Hortelano et al. [28], which was a watchdog-based intrusion detection system (IDS). In this scheme, when a source node A transmits packets to an intermediate node B, then node A checks whether node B further forwarded the packets or not to the next vehicle by continuously listening to node B’s transmission. Every node maintains a table of its neighbors’ trust levels. If a malicious node drops the packets repeatedly and exceeds the threshold level, then that node is declared malicious. The scheme has proven to be effective in detecting selfish and malicious vehicles. However, due to the periodic listening of nodes’ actions and maintaining an extra buffer for recording other nodes’ trust levels, the scheme generates an additional routing overhead and end-to-end delay. Similarly, in [29], Daeinabi et al. proposed an algorithm for the detection and isolation of malicious vehicles in VANET called DMV (detecting malicious vehicle). In this algorithm, vehicles are grouped into clusters led by a cluster head (CH). Whenever a new vehicle enters the cluster, the verifier vehicle starts scanning the entered vehicle’s actions. If the entered vehicle continuously drops the packets, then the verifier vehicle reports it to the CH. The CH decreases the reported vehicle’s trust value. If the trust value of the reported vehicle reaches a pre-defined threshold, then CH reports it to the certification authority (CA). The CA then enters it into the blacklist and informs all other vehicles through alarms. The simulation results show that the proposed approach is capable of detecting most of the available attacks in VANETs. However, the approach takes longer to process and has an impact on other performance metrics, including throughput, end-to-end delay, and jitter [30].

In [30], Kadam et al. proposed the detection and prevention of malicious vehicles (D&PMV) to address BHAs in VANETs. The authors made some improvements to the DMV algorithm proposed in [29] by adding the cache mechanism for path construction during the route discovery phase. This algorithm first scans all the existing paths for the availability of BHA; if the path with a BHA is found, then it ignores the path and reconstructs a new path. As compared to DMV, this algorithm can detect and prevent BHAs with high mobility and reduce the impact of BHAs inside VANETs. However, this algorithm still requires additional time for its processing, which results in high end-to-end delay [20]. In [31], Dhaka et al. proposed a scheme for the identification and removal of BHAs and GHAs. The authors modified the original AODV routing protocol by adding two additional control packets, i.e., the response sequence (Rseq) and the code sequence (Cseq). In this scheme, a source node broadcasts the Cseq packet to all of its neighboring nodes. Upon receiving the Cseq, each node responds with the Rseq packet. A connection is established toward the destination if both packets’ IDs match a specific neighbor. Otherwise, the source node discards the Rseq of the node and informs all other nodes about the malicious node. The scheme provides a higher PDR and is applicable in other reactive routing protocols. However, due to the usage of additional control packets, the technique causes substantial routing overhead in the network.

In [32], Jahan and Suman proposed an acknowledgment-based model to detect BHA in VANETs. In this model, each intermediate node informs the source node through an acknowledgment that it has forwarded the packet to the next-hop node. This process is continued until the destination is reached. This model generates excessive network congestion due to the use of extra acknowledgments provided by each intermediary node, causes substantial routing overhead, affects the PDR, and generates delay. In [33], Li et al. proposed an attack-resistant trust (ART) management scheme based on evaluating the trustworthiness of data and nodes to identify and detect malicious nodes. The scheme is split into two phases: data analysis and trust management. First, the traffic data is collected from vehicles and then analyzed using Dempster–Shafer theory. However, it is possible that some malicious nodes forward packets correctly but later start acting maliciously (i.e., dropping data packets).

In [34], Purohit et al. proposed a secure vehicular on-demand routing (SVODR) scheme to mitigate BHAs in VANETs. A new field called an encrypted random number is inserted into the RREQ packet and broadcast to all its neighboring nodes. Upon receiving the RREP, the source node checks its own routing table’s destination sequence number (DSN) and the RREP’s DSN and encrypted/decrypted random numbers. A node is genuine if its RREP’s DSN is greater than the source vehicle’s routing table DSN and both functions’ random numbers are equal. Otherwise, the vehicle is declared malicious. A downside of this scheme is that it requires extra fields in the control packets for cryptographic algorithms that need extra resources, resulting in a large routing overhead and end-to-end delay. In [35], Tyagi et al. proposed a three-step BHA detection algorithm called enhanced secure AODV (ES-AODV). In step 1, the RSU plays an additional role as the certificate authority (CA), which manages public and private key pairs. In step 2, the source broadcasts the RREQ packet along with the vehicle’s certificate, nonce encryption, and the public key of the destination. In step 3, a BHA is detected based on the threshold value obtained from the sequence number of RREP and verification of the nonce value. The technique is built on public-key cryptography, which protects the network against external attacks, but an internal BHA may create disruption. Second, to detect a BHA, the method requires the presence of RSUs, which may not be applicable in all VANET scenarios.

In [36], Zardari et al. proposed a dual-attack detection of BHA and GHA (DDBG) scheme based on a connected dominating set (CDS) and IDS to detect malicious nodes. In this scheme, the IDS node broadcasts a status packet and starts waiting for its response. On receiving all the replies, the IDS node checks which node has not sent a reply properly and why. If any node does not respond or sends a bogus reply, that node is declared as a malicious node. The key problem with this scheme is that it periodically broadcasts a status packet to detect malicious nodes in the network, which results in a huge routing overhead. In [4], Cherkaoui et al. proposed a novel method to detect BHAs in VANETs based on using a variable control chart. The method is implemented in each receiving vehicle to detect the BHA through the supervision of the throughput and end-to-end delay metrics. Each vehicle calculates the parameters of the chart and transforms the received packets into a graphical representation. A node is declared malicious when the metrics curves oscillate outside of the chart limits. However, deploying the monitoring system on each receiving individual node causes unnecessary processing overload. Second, the techniques are often used in industrial fields to monitor the quality of a particular system; therefore, using a variable control chart in the VANET context is impracticable.

In [20], Hassan et al. proposed an intelligent detection BHA (IDBA) scheme in autonomous and connected vehicles (ACVs). The scheme pre-calculates four threshold values from the four key metrics: sequence number, hop count, PDR, and end-to-end delay (i.e., Th1, Th2, Th3, and Th4, respectively). According to this scheme, when a node receives a new RREP packet, it checks whether the RREP’s sequence number is greater than Th1 and the hops count is equal to Th2; if so, it adds such a node into the gray-list. Then, the node checks whether the PDR is greater than Th3 and the end-to-end delay is less than Th4; if so, the gray-listed node is assigned to the black-list. An alarm message is flooded into the network to isolate the BHA node. The scheme is completely based on pre-calculated threshold values generated from old data so that the traffic condition, such as congestion, may be changed from time to time. Thus, threshold values generated in advance may consider a malicious node genuine and vice versa. In addition to that, calculating four key thresholds on each node results in high end-to-end delay and processing overhead. In [10], Kumar et al. proposed a secure AODV (SAODV) with improvements made in the RREQ and RREP control packets. To detect a BHA, first, a message is forwarded to the neighboring nodes to know their status. Second, an encrypted packet is forwarded to all its neighboring nodes to calculate their reputation. Third, the forwarded packets are verified for reputation. Fourth, a secret key is forwarded to the known neighbors. Finally, the RREQ and RREP are verified and start forwarding data packets. The source node appends an encrypted value (sequence number) in the RREQ and broadcasts it to all the neighboring vehicles. On receiving the RREP, the source node declares a node as malicious if the encrypted value of the routing table and the decrypted value of the RREP are not equal. The approach uses extra fields in the control packets for cryptographic functions, which needs extra resources, resulting in a heavy routing overhead. Second, it contains five different phases to identify and detect BHA, which is quite complex and generates extra processing overhead, resulting in high end-to-end delay.

The details, pros, and cons of each of these schemes are given in Table 1. In VANETs, a BHA is a major security threat in which a malicious node drops all the data packets and does not forward them to other nodes in routing, which leads to degradation of the overall security and performance of the VANET. To stop this attack, many solutions are presented in the literature. From the critical analysis of the related literature shown in Table 1 above, it is evident that the existing schemes have many limitations. For example, most of these schemes [8,20,28,29,30,31,32,34,36] employed some extra DPS/IDS nodes and exchanged additional control packets, which increased the routing overhead and end-to-end delay. The PDR decreases whenever the network is denser, and the higher the end-to-end delay in the network leads to lower average throughput. These limitations cause the consumption of valuable network bandwidth and compromise network performance and security. To address these challenges, we present a novel solution for detecting and preventing a BHA with a small routing overhead and end-to-end delay in this study. Furthermore, the proposed solution improves VANET security and performance by increasing the PDR and throughput while eliminating false positive and false negative rates. The proposed solution used a new approach based on calculating a dynamic threshold value from sequence numbers and generating a forged RREQ packet.

## 4. Proposed Work

**Figure 4:**
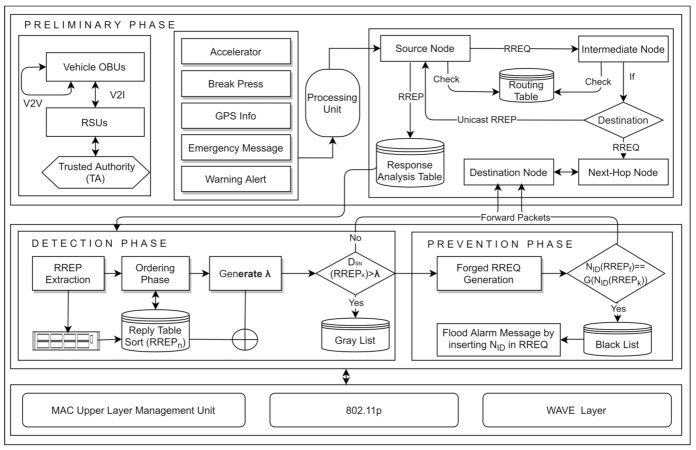
The framework of DPBHA.

In this section, we elaborate and discuss the proposed detection and presentation of a black hole attack (DPBHA). The proposed DPBHA exploits the two main malicious properties of a BHA. First, the RREP of the attacker node contains a higher sequence number and minimum hop count value since it pretends to have a fresh route toward the destination. Second, the attacker node always responds first to every RREQ without going to check its routing table. Fair modifications are made in the default operations of the AODV routing protocol to take advantage of these two properties to detect and prevent BHAs in VANETs. The proposed DPBHA operates mainly in three phases, i.e., the connectivity phase, detection phase, and prevention phase, as shown in Figure 4.

In the connectivity phase, the network under consideration is initiated, the topology is established and communication between vehicles (nodes) is assumed to be started. The suspected malicious node that tends to be a black hole (with a 50% likelihood) is found in the second phase. The suspected malicious node is 100% proven to be a black hole node in the third phase, and it should be removed from the network.

### 4.1. Connectivity Phase

A highly dynamic VANET in which N number of nodes (vehicles and RSUs) are randomly deployed across the road segment in an urban traffic area. All vehicles are assumed to be intelligent, i.e., embedded with onboard units (OBUs). Each vehicle’s OBU has radio equipment, such as a global positioning system (GPS) for location tracking and IEEE 802.11p for communication purposes. Furthermore, RSUs are deployed along roadsides at equal distances to cover the urban traffic area. In traffic management theory, the free-flow state denotes low traffic density and weak vehicle interaction. We investigated the connectivity of VANETs in the free flow state in this research work. According to empirical studies, the Poisson distribution is an excellent model for the vehicle arrival rate in the free-flow state [37,38]. The speeds of different vehicles in a free-flow state follow a normal distribution [39,40]. We suppose that each vehicle is given a random speed from a normal distribution and maintains that speed while traveling on the highway.

**Figure 5:**
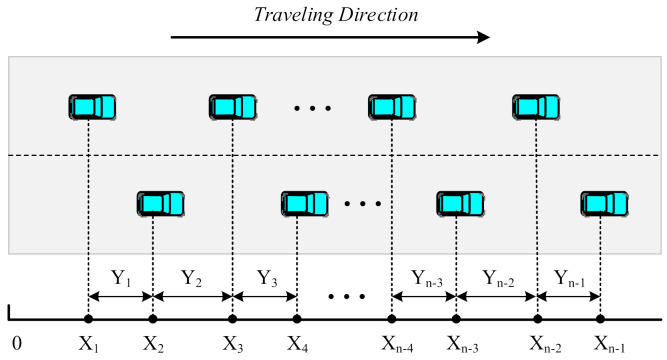
The mobility model of vehicles.

Graph theory is a promising approach for modeling and representing the connectivity analysis of vehicular networks [41,42]. A random geometric graph (RGG) is a particular model of traditional graph theory that accurately characterizes randomly deployed networks, such as wireless sensor networks [43,44,45,46,47] or VANETs [48]. In an RGG, the nodes are independently distributed at random according to some spatial probability distribution, and two nodes can be connected by an edge if and only if the distance between them is less than the transmission range ($T_{R}$). The topology of a VANET is represented by an RGG, where nodes in such a graph are independently deployed according to a Poisson distribution with a transmission range $T_{R}\geq 0$ [49]. Let us assume that a graph$\;G=\left(N,\;E,\;C\right)$, where N indicates a set of nodes (vehicles and RSUs), E represents a set of edges (links), and C represents a set of connections among nodes. The graphical representation of VANET’s topology is given by Equation (1).

(1) $$A=\left\{\begin{array}{ll} C_{\mathrm{ij}}, & \mathrm{If}\;(V_{i}\;,{\;V}_{j})\;\in \;C\;\mathrm{and}\;0\;<\;C_{\mathrm{ij}}\;<\;1\\ 1, & \mathrm{if}\;\;\;i=j\\ 0, & \mathrm{otherwise} \end{array}\right.$$

where A is the affinity matrix and $(V_{i}\;,{\;V}_{j})\;\epsilon \;C$ signifies that $V_{i}\;$and $V_{j}$ are connected. To compute the vehicular network’s adjacency matrix, we employed three conditions:

(1) If vehicles $V_{i}$ and $V_{j}$ are connected, the value of the link connectivity is added to the ijth position of the adjacency matrix Adj.
(2) If a link $C_{\mathrm{ij}\;}$ has the same connectivity in both directions $\left(i=j\right)$, 1 is added to the connectivity. However, a node can be connected to itself through other nodes in a multi-hop manner, for instance, V_1_→V_3_→V_4_→V_1_.
(3) When the above two conditions fail, the term “otherwise” is evaluated in Equation (1). When two vehicles are not connected, we add zero. The adjacency matrix Adj, which represents vehicle interconnectivity, is given by Equation (2).

(2) $$\mathrm{Adj}=\left[\begin{array}{ccccc} C_{11} & C_{12} & C_{13} & \ldots & C_{1n}\\ C_{21} & C_{22} & C_{23} & \ldots & C_{2n}\\ \vdots & \vdots & \vdots & \ddots & \vdots \\ C_{n1} & C_{n2} & C_{n3} & \ldots & C_{\mathrm{nn}} \end{array}\right]\;$$

where C denotes the connection reliability between two vehicles. Suppose a segment of a unidirectional two-lane highway of length L kilometers is labeled by interval $M=\left[0,L\right]$. Each node enters the highway at X=0 with a random speed and exits at X=L. We assumed that the process of vehicles entering the highway follows a Poisson distribution. As shown in Figure 5, $X_{i}$ denotes the location of the ith vehicle from the origin and the headway is represented as $Y_{i}=X_{i+1}-X_{i}$ and $Y_{0}=X_{1}$ for i=1, 2, 3, …, n−1.

If a vehicle $V_{i}$ is lying within the transmission range $\left(T_{R}\right)$ of another vehicle $V_{j\;}$, i.e., $\left(({\mathrm{distance}\;\mathrm{between}\;V}_{i}{\;\mathrm{and}\;V}_{j\;}\right)\leq T_{R\;}$), then they are presumed to be connected by a unidirectional link $l_{i\;}\epsilon \;E.$ Whenever $V_{i\;}$ transmits a packet, it is directly received by $V_{j}$ via an edge $l_{i}$. An edge $E=({\;V}_{i},{\;V}_{j\;})$ exists between two vehicles if the Euclidean distance [50] between them is less than or equal to their $T_{R}$, as given in Equation (3).

(3) $$E=\left\{({\;V}_{i},{\;V}_{j\;})|({\;\mathrm{POS}}_{i}-{\;\mathrm{POS}}_{j})\leq T_{R}\right\}$$

where ${\mathrm{POS}}_{i}$ and ${\mathrm{POS}}_{j}$ are the coordinates for vehicle $V_{i}$. and vehicle $V_{j}$, denoted by $\left(X_{i},Y_{i}\right)$ and $\left(X_{j},Y_{j}\right)$, respectively at time${\;t}_{0}$. The equation leads to an undirected graph that may be connected or unconnected based on the Euclidean distance (d) between $V_{i}$ and $V_{j}$, as calculated using Equation (4).

(4) $$d=\sqrt{{\left(X_{i}-X_{j}\right)}^{2}+{\left(Y_{i}-Y_{j}\right)}^{2}}$$

If the distance between two nodes is greater than their transmission range, then the packets are exchanged between them indirectly in a multi-hop fashion. Consider $\left(X_{s},Y_{s}\right)$ and $\left(X_{{\;N}_{N}},Y_{{\;N}_{N}}\right)$ as the coordinates of a source node S and a neighboring node $N_{N}$, respectively, with their corresponding speeds denoted by $V_{s}$ and $V_{N_{N}},$ respectively, and $T_{R}$ is the transmission range. Therefore, the link (E) lifetime between the S and${\;N}_{N}$ nodes are calculated using Equation (5).

(5) $$E_{s,N_{N}}=\frac{T_{R}-\sqrt{{\left(X_{{\;N}_{N}}-X_{s}\right)}^{2}+{\left(Y_{{\;N}_{N}}-Y_{s}\right)}^{2}}}{V_{s}-{\;V}_{N_{N}}\;}$$

Assume that there are N number of nodes, which are randomly distributed in an urban area of w×l square meters, $T_{R}$ is the transmission range, S is the source, and D is the destination node. The probability $\left(P\right)$ of a neighboring node $N_{N}$ being within the transmission range of node S is calculated using Equation (6).

(6) $$P=\frac{{\pi \;T}_{R}{}^{2}}{w\times l}$$

The two most important metrics for measuring the performance of highly dynamic networks are link reliability [40] and connectivity [38]. The truncated Gaussian probability density function (PDF) of the vehicle’s velocity is given by Equation (7).

(7) $$f_{v}\left(v\right)=\frac{1}{\sigma \sqrt{2\pi }}e^{-\left(\frac{{\left(v-µ\right)}^{2}}{2\sigma^{2}}\right)}$$

where µ is the average speed and σ is the standard deviation of the vehicle speed. On the road segment, two vehicles are said to be connected if and only if they are lying within each other’s transmission range$\;\left(T_{R}\right)$. Vehicle connection is determined by the generalized speed factor (GSF) in [38], which indicates the number of vehicles on a certain road segment in units of km/h and the effect of relative velocity with inter-vehicle spacing. The normal distribution of relative speed and the exponential distribution of inter-vehicle spacing are used to define the GSF [38,39]. Therefore, the definition of the GSF is a truncated Gaussian PDF [39], as given by Equation (8).

(8) GSF =∫vminvmax fv^vv dv

where

(9) fv^v=fvv∫vminvmax1µe−−sµds

where $f_{v}\left(v\right)$ is the Gaussian PDF of the vehicle’s velocity defined in Equation (7), $v_{\min}$ is the minimum speed, and $v_{\max}$ is the maximum speed of a vehicle. Moreover, v denotes the speed and s denotes the inter-vehicle spacing, where they have an indirectly proportional relationship to each other. According to the definition of the GSF, the probability of the connectivity of N number of vehicles at time can be obtained using Equation (10).

(10) $$P_{c}\left(N\right)t=\overset{N-1}{\underset{i=1}{\prod }}\left(1-e^{-\left(p\right)\left(\mathrm{GSF}\right)\left(T_{R}\right)\;}\;\right)={\left(1-e^{-\left(p\right)\left(\mathrm{GSF}\right)\left(T_{R}\right)\;}\right)}^{N-1}\;$$

where p denotes the density of vehicles and $T_{R}$ is the V2V transmission range. Equation (10) indicates that the speed, density, and transmission range of inter-vehicle communication significantly affects the vehicle connectivity process on a free-flow highway. The notations used in this paper and their descriptions are tabulated in Table 2.

**Assumptions**—For the development of our proposed DPBHA and its operations to work, some assumptions were necessary in order to provide a consistent scenario within which to work. These assumptions are reasonable and useful to consider in accordance with the design consideration of VANETs. These assumptions are:

(1) We assumed that the black hole node is a malicious node that always exploits its harmful properties to each requesting node and that all other nodes are genuine nodes that act normally.
(2) All the network nodes should be uniquely identifiable, and only BHA will exist in the network. Other network attacks, such as a GHA, Sybil attack, or impersonation attack, will not exist.
(3) The solution assumed that multiple RREPs will arrive at the source node during the route discovery process and they will be stored in an additional response analysis table (RAT).
(4) All the network nodes have the same features, and it was assumed that if node A is lying in the transmission range of node B, then node B will also lie in the transmission range of node A.
(5) All the nodes were assumed to be healthy and they must participate in the route discovery process according to assumption (1).

### 4.2. Detection Phase

In this phase, a dynamic threshold value is generated to identify the malicious node (black hole node) in the network. Upon receiving all possible RREPs within a time t $\left(\mathrm{rrep}\_\mathrm{time}\_\mathrm{out}\right),\;$the source node stores them in the RAT. To calculate the threshold value$\;\left(\lambda \right),$ the source node sorts out all the received RREPs in descending order with respect to destination sequence number ($D_{\mathrm{SN}}$). Then, S calculates the average of all the received RREPs’ $D_{\mathrm{SN}}$ values with the difference of the last RREP’s $D_{\mathrm{SN}}$ from its routing table’s $D_{\mathrm{SN}}.$ The calculation procedure of the λ is presented in the following Equation (11).

$$\lambda \;=\;\mathrm{Average}\left[\begin{array}{c} \left(D_{\mathrm{SN}}\left({\mathrm{RREP}}_{1})-\left(D_{\mathrm{SN}}({\mathrm{RREP}}_{n}\right)-D_{\mathrm{SN}}({\;R}_{T}\right))\right)\\ +\left(D_{\mathrm{SN}}\left({\mathrm{RREP}}_{2})-(D_{\mathrm{SN}}({\mathrm{RREP}}_{n})-D_{\mathrm{SN}}\left({\;R}_{T}\right)\right)\right)+\\ \left(D_{\mathrm{SN}}\left({\mathrm{RREP}}_{3})-(D_{\mathrm{SN}}({\mathrm{RREP}}_{n})-D_{\mathrm{SN}}\left({\;R}_{T}\right)\right)\right)+\ldots \\ +\left(D_{\mathrm{SN}}\left({\mathrm{RREP}}_{n})-(D_{\mathrm{SN}}({\mathrm{RREP}}_{n})-D_{\mathrm{SN}}\left({\;R}_{T}\right)\right)\right) \end{array}\right]+\;n\left(D_{\mathrm{SN}}({\mathrm{RREP}}_{n})-D_{\mathrm{SN}}(R_{T})\right)$$

(11) $$=\frac{\sum_{i=1}^{n}D_{\mathrm{SN}}\left({\mathrm{RREP}}_{i})-\left(D_{\mathrm{SN}}({\mathrm{RREP}}_{n}\right)-D_{\mathrm{SN}}({\;R}_{T}\right))}{n}+n\left(D_{\mathrm{SN}}({\mathrm{RREP}}_{n}\right)-D_{\mathrm{SN}}\left({\;R}_{T}\right))$$

The difference between the last RREP’s $D_{\mathrm{SN}}$ and its routing table’s $D_{\mathrm{SN}}$ is calculated using (12).

(12) $$\Delta =D_{\mathrm{SN}}({\mathrm{RREP}}_{n})-D_{\mathrm{SN}}\left({\;R}_{T}\right)$$

where ∆ denotes the difference between the sequence number of the last RREP and existing${\;R}_{T}$. To further simplify the above formula for calculating the threshold value $\left(\lambda \right)$, the equation can be written as Equation (13).

(13) $$\lambda =\left|µ\left(\overset{n}{\underset{i=1}{\sum }}D_{\mathrm{SN}}\left({\mathrm{RREP}}_{i}\right)-\Delta \right)+n\Delta \right|\;$$

The source node checks each RREP’s $D_{\mathrm{SN}}$ with the calculated threshold value $\left(\lambda \right)$ shown in Equation (14). The RREP with a higher $D_{\mathrm{SN}}$ than the threshold value $\left(\lambda \right)$ will be considered as a malicious node.

(14) $$N_{\mathrm{ID}}\;\left({\mathrm{RREP}}_{k}\right)=\left\{\begin{array}{c} G,\;\;\mathrm{if}\;\left(D_{\mathrm{SN}}\left({\mathrm{RREP}}_{k}\right)>\lambda^{\mathrm{th}}\right),\\ N,\;\;\mathrm{otherwise} \end{array}\right.$$

**Figure 6:**
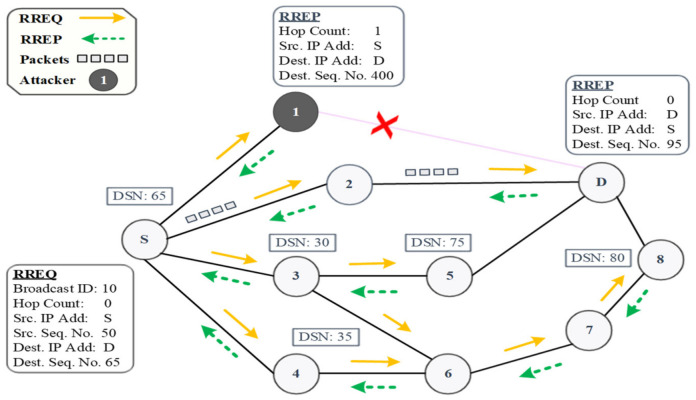
A scenario demonstrating the detection phase.

Figure 6 illustrates an experimental scenario of the detection phase. In this experiment, we assumed that node S is the source node, node D is the destination node, node 1 is the black hole attacker node, and all the remaining nodes are intermediate nodes.

After broadcasting an RREQ packet, node S receives four RREP packets and sorts them in descending order with respect to $D_{\mathrm{SN}}$ in its RAT, as shown in Table 3. To calculate the threshold value$\;\left(\lambda \right)$, first, node S calculates the difference (Δ) between the last RREP’s $D_{\mathrm{SN}}$ and its routing table’s $D_{\mathrm{SN}}$ by putting the values into Equation (12), which gives Δ = 75 − 65 = 10. Now, node S calculates the threshold value from all the received RREPs by putting the values into Equation (13), i.e.,$\;\lambda \;=\;\left(\left(390\;+\;85\;+\;70\;+\;65\right)/4\right)\;+\;40=193$. Next, node S compares each received RREP’s $D_{\mathrm{SN}}$ value with λ. Node S finds that node 1 has a higher $D_{\mathrm{SN}}$ (400) than the threshold value (λ = 193). Node S marks it as a suspicious node with a 50% probability and moves it into the gray list.

Furthermore, to confirm whether the suspected node that claims a higher $D_{\mathrm{SN}}$ is really malicious or it is a genuine node, the source node pledges to the next phase. 

### 4.3. Prevention Phase

In this phase, the source node modifies the format of the RREQ packet by replacing a non-existing IP address over the destination node IP address field. The new forged RREQ packet format is shown in Table 4. The source node broadcasts the forged RREQ packet in the network. Only a malicious node can give a response, as it does not search the routing table for the route toward the destination and produces an RREP packet. If the same node that is marked as a 50% suspected in the previous phase responds to a forged RREQ, then that particular suspicious node will be confirmed and marked as a 100% black hole node, shown in Equation (15). The source node immediately enlists it to the black list and broadcasts the alarm message into the network by inserting the identity of the black hole node in the RREQ.

(15) $$f\left(N_{\mathrm{ID}},\left({\mathrm{RREP}}_{k}\right)\right)\;=\;\left\{\begin{array}{c} G\leftarrow \left[N_{\mathrm{ID}}\right],\;{\;\mathrm{if}\;D}_{\mathrm{SN}}\left({\mathrm{RREP}}_{k}\right)\;>\lambda^{\mathrm{th}}\;\\ \;\;\;\;\;S\;\to \;{\mathrm{RREQ}}_{\mathrm{forged}}\\ B\leftarrow G,\;\;\;\;\;\mathrm{if}\;\left(N_{\mathrm{ID}\left({\mathrm{RREP}}_{f}\right)}=\;G\left(N_{\mathrm{ID}\left({\mathrm{RREP}}_{k}\right)}\right)\right)\\ \;\;\;\;\;S\;\to \;{\mathrm{Alarm}}_{\mathrm{message}\;}\mathrm{to}\;N_{N}\\ N\leftarrow G,\;\;\;\;\;\mathrm{Otherwise};\\ \;\;\;\;\;S\;\to \mathrm{Packets}\;\mathrm{to}\;D \end{array}\right.$$

**Figure 7:**
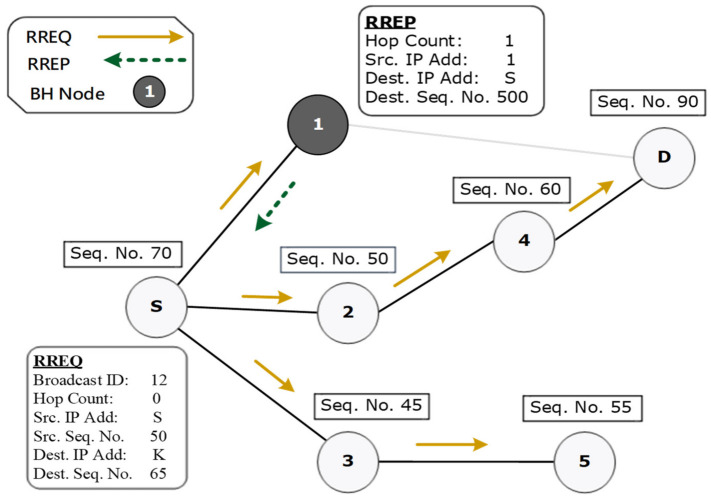
A scenario demonstrating the prevention phase.

**Figure 8:**
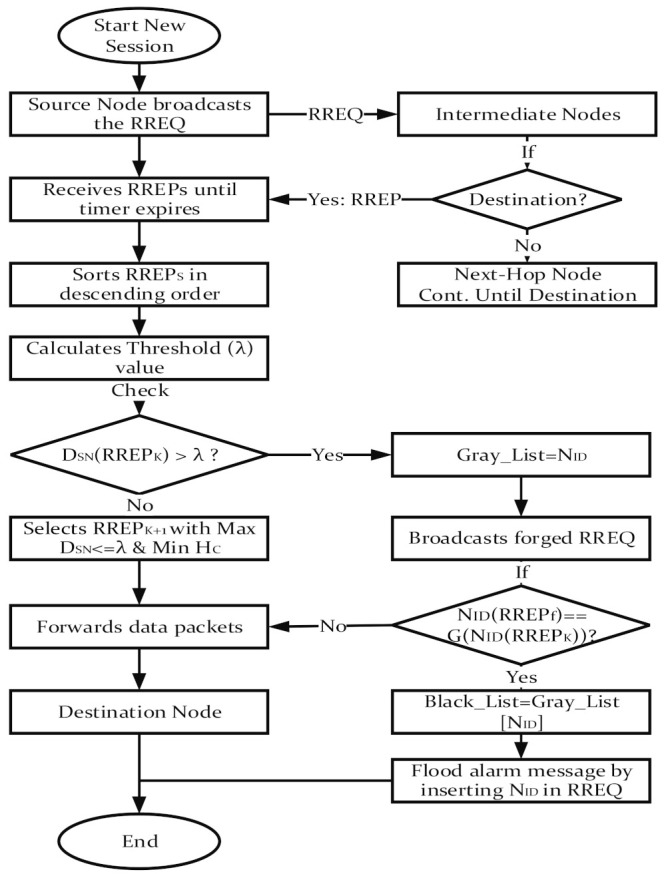
Flowchart of the proposed DPBHA.

The next RREP’s route with the highest $D_{\mathrm{SN}}$ below or equal to the threshold value and minimum hop count will be selected for routing data packets. Figure 7 depicts an experimental scenario of the prevention phase with the generation of a forged RREQ packet. In Figure 7, the source node broadcasts the forged RREQ with a destination IP address K in the network. Here, a genuine node will not reply as the forged RREQ has an IP address that does not exist in the network. Only a malicious node can give a response, as it does not search the routing table for the route toward the destination; therefore, node 1 unicasts the RREP. Upon receiving the RREP, the source node confirms and marks it as a black hole node. Figure 8 illustrates a complete flowchart for the proposed DPBHA, along with the internal data flow processes of the three core phases. Algorithm 1 illustrates the complete step-by-step process of the proposed DPBHA solution.


**Algorithm 1:** Black Hole Attack Detection and Prevention **Input:** RREQ, RREP, G, B, Forged-RREQ **Output:** BHA Detection and Prevention, Best and Secure Path Selection**1.**  
**Initialization:**

i=0, 1, 2, 3,…, n

**2.** 

$S\;\to {\;\mathrm{RREQ}\;\mathrm{to}\;N}_{N}\;\mathrm{and}\;\mathrm{sets}\;t$

**3.** 

$if{\;\mathrm{route}\;\mathrm{to}\;D\;\mathrm{in}\;R}_{T}$


**4.**
   

goto step 11


**5.**


else


**6.**
  

do 


**7.**
   

$N_{N}\to {\;\mathrm{RREQ}\;\mathrm{to}\;N}_{\mathrm{HN}}$


**8.**
   

$while\left(N_{N}=D\right)$


**9.**
  

end


**10.**


end


**11.**


${D\;v\;N}_{N}\to \;\mathrm{RREP}\;\mathrm{to}\;S$


**12.**


$S\;\left[\mathrm{RAT}\right]\;\leftarrow {\;\mathrm{RREP}}_{i}\;\mathrm{till}\;t$


**13.**


$\mathrm{Quicksort}\left(S\;\left[\mathrm{RAT}\left(D_{\mathrm{SN}}\left({\mathrm{RREP}}_{i}\right)\right)\right],\;\mathrm{start},\;\mathrm{end},\;\mathrm{pivot}\right)$


**14.**


$\Delta =D_{\mathrm{SN}}({\mathrm{RREP}}_{n})-D_{\mathrm{SN}}\left({\;R}_{T}\right)$


**15.**


$\lambda =\left|µ\left(\overset{n}{\underset{i=1}{\sum }}D_{\mathrm{SN}}\left({\mathrm{RREP}}_{i}\right)-\Delta \right)+n\Delta \right|$


**16.**


$\forall {\;\mathrm{each}\;\mathrm{RREP}}_{i-n}\in \;\left[\mathrm{RAT}\right]\;$


**17.**


$if\;(D_{\mathrm{SN}}\left({\mathrm{RREP}}_{k}\right)>\lambda^{\mathrm{th}})$


**18.**
  

$G\;\leftarrow {\;N}_{\mathrm{ID}}\;\left({\mathrm{RREP}}_{k}\right)$


**19.**


else


**20.**
  

${\mathrm{Selects}\;\mathrm{RREP}}_{k+1}({\mathrm{Max}\;D}_{\mathrm{SN}}<={\;\lambda }^{\mathrm{th}}\;\mathrm{and}\;\mathrm{Min}\;\mathrm{HC})\;$


**21.**
  

goto step 30


**22.**


end


**23.**


$S\;\to {\;\mathrm{RREQ}}_{\mathrm{forged}}{\;\mathrm{to}\;N}_{N}$


**24.**


$S\leftarrow {\;\mathrm{RREP}}_{f}$


**25.**


$if\;\left(N_{\mathrm{ID}\left({\mathrm{RREP}}_{f}\right)}=\;G\left(N_{\mathrm{ID}\left({\mathrm{RREP}}_{k}\right)}\right)\right)$


**26.**
  

$B\;\leftarrow \;G\;\left[N_{\mathrm{ID}}\right]$


**27.**
  

$S\;\to {\;\mathrm{Alarm}}_{\mathrm{message}\;}{\mathrm{to}\;N}_{N}$


**28.**
  

goto step 20


**29.**


else


**30.**
  

S →Packets to D


**31.**


end




## 5. Implementation and Result Evaluation

The proposed DPBHA was implemented and evaluated in a simulation-based environment (NS-2 Simulator v2.35) and its performance and efficacy were compared to the benchmark schemes. NS-2 allows for a wide range of simulation settings, making simulation more practical and realistic. The results were compared with the most relevant schemes that exist in the literature, namely, AODV [19], SAODV [10], and IDBA [20]. The parameters used in the simulation experiments are tabulated in Table 5.

For the performance evaluation, a general urban traffic scenario was selected with a variable traffic density of 25 to 150 nodes (vehicles, RSUs, and black hole nodes). Each simulation experiment contained 8% malicious nodes (black hole nodes). 

**Figure 9:**
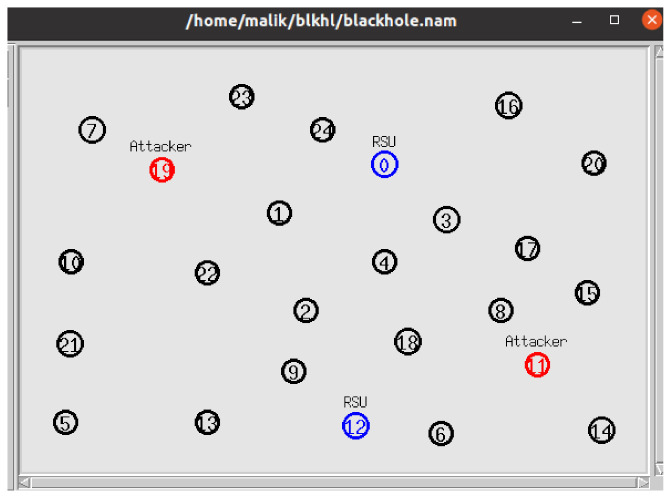
Initial state of the first experiment.

Figure 9 demonstrates one of the initial states of the first experiment performed with 25 nodes comprising 21 normal vehicles (with black circles), 2 black hole nodes (with red circles), and 2 RSUs (with blue circles). Before performing the statistical analysis, each simulation experiment was run 10 times in the simulator and the average values were obtained after aggregating the results. The following performance metrics were used to evaluate the proposed solution:

- Routing overhead;
- Packet delivery ratio (PDR);
- End-to-end delay;
- Throughput;
- Packet loss ratio;
- Confusion metrics.

### 5.1. Routing Overhead

The routing overhead (ROH) represents the ratio of the total number of control packets transmitted to the total number of data packets, as given in Equation (16).

(16) $$\mathrm{ROH}=\frac{\sum \mathrm{control}\;\mathrm{packets}\;\mathrm{transmitted}}{\sum \mathrm{data}\;\mathrm{packetstransmitted}}$$

**Figure 10:**
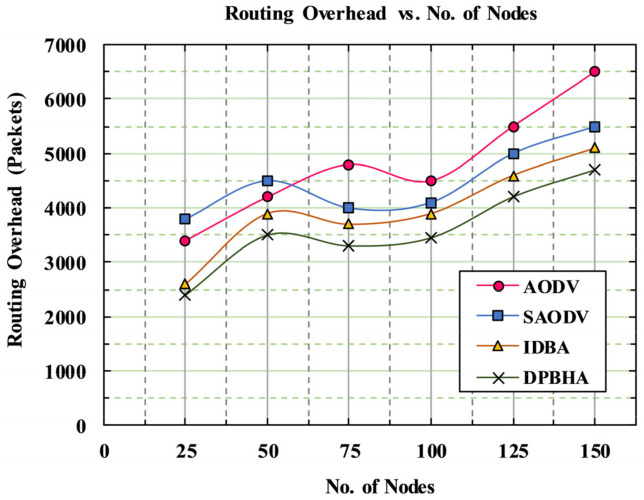
Graphical representation of routing overhead.

Figure 10 shows the simulation results, indicating the number of nodes on the *x*-axis and the routing overhead (in the number of packets) on the *y*-axis. The routing overhead increased with respect to an increase in the number of nodes. As the network became more congested, path breakages and packet drop rates became more common. The presence of more malicious nodes caused more RREPs to be sent to the desired route, resulting in increased routing overhead. The routing overhead behavior for the proposed DPBHA was plotted in comparison to benchmark schemes, namely, classic AODV, SAODV, and IDBA. By detecting the malicious nodes instantaneously from the network, the routing overhead was reduced in the proposed DPBHA as compared to the benchmark schemes. In the case of classic AODV, more replies were generated in the network due to the presence of malicious nodes, resulting in a huge routing overhead of 28.57%. Similarly, in the case of SAODV, more control packets were generated in its five-step detection mechanism such that its routing overhead was 26.59%, which was also very high. In the case of IDBA, the average routing overhead was 23.52% which was close to the proposed DPBHA. Figure 10 indicates that in most of the points in DPBHA, the average routing overhead was 21.30%, which was the minimum among all the schemes. Therefore, the proposed DPBHA decreased the average routing overhead by 3.69%.

### 5.2. Packet Delivery Ratio

The packet delivery ratio (PDR) represents the ratio of the total number of packets received at a destination node to the total number of packets originated at the source node, as shown in Equation (17).

(17) $$\mathrm{PDR}=\frac{\sum \mathrm{Number}\;\mathrm{of}\;\mathrm{packets}\;\mathrm{received}}{\sum \mathrm{Number}\;\mathrm{of}\;\mathrm{packets}\;\mathrm{sent}\;}$$

**Figure 11:**
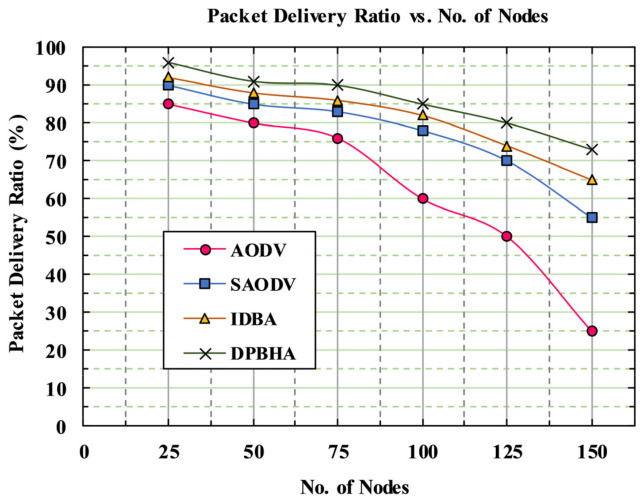
Graphical representation of packet delivery ratio.

Figure 11 shows the simulation results, indicating the PDR in terms of percentage on the *y*-axis and the number of nodes on the *x*-axis. It can be observed that as the number of nodes increased, the PDR decreased due to the presence of more malicious nodes and packet collision occurrences in the network. When a malicious node performs a packet-dropping attack, it badly affects the PDR. The proposed DPBHA first identifies the malicious node with the help of a dynamic threshold value and then confirms it as malicious by broadcasting a forged RREQ.

In Figure 11, it can be observed that the proposed DPBHA had the best performance results in PDR compared to the rest of the schemes. The PDR decreased significantly in the case of the classic AODV, with an average of 20.44%, while the PDR of other schemes showed less of a decrease due to the presence of some security mechanisms. The average PDRs for the SAODV and IDBA schemes were recorded as 25.06% and 26.48%, respectively. The classic AODV severely suffered from the presence of a BHA: as the number of malicious nodes in the network grew, its PDR dropped drastically. The average PDR of our proposed DPBHA was 28%, which was a 3.0% improvement above the total average PDR.

### 5.3. Throughput

Throughput represents the average rate of successful data packet delivery to the final destination by the source node, as given in Equation (18). Throughput can be measured in packets per second (pps), bits per second (bps), or packets per time slot.

(18) $$\mathrm{Throughput}=\frac{\sum \left(\mathrm{Received}\;\mathrm{Packets}*\mathrm{Packet}\;\mathrm{Size}\right)\;}{\mathrm{Simulation}\;\mathrm{Time}}$$

**Figure 12:**
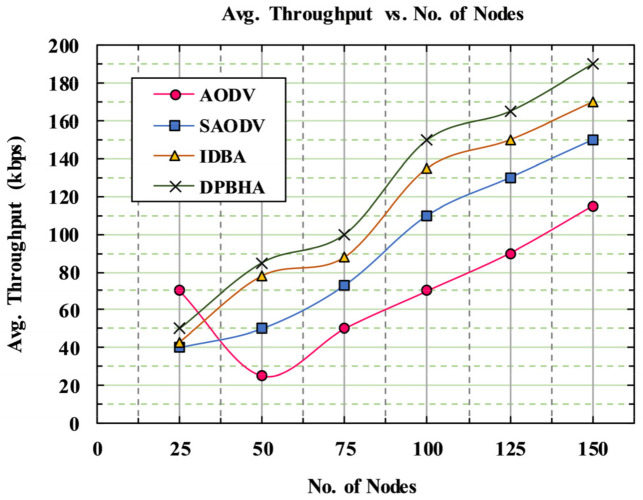
Graphical representation of average throughput.

Figure 12 shows the performance of the throughput metric (in kbps) for the proposed DPBHA and benchmark schemes. The throughput of the classic AODV had the lowest significant values on each point because of the presence of BHAs and the destination node received extremely few packets. Another reason for the throughput degradation was the high speed of the vehicles, causing frequent link breakages, which led to a decrease in throughput. The average throughput of the classic AODV was recorded as 17.68%, which drastically suffered from the increase in the number of malicious nodes in the network. The average throughputs of the SADOV and IDBA schemes were recorded as 23.36% and 27.78%, respectively. These schemes achieved a certain level of better performance in throughput because both of them employed some security mechanisms that detect a BHA instantly. In terms of throughput, the proposed DPBHA outperformed the existing schemes. The average throughput of the proposed DPBHA was recorded as 31.15%, which was the highest among all the schemes. Therefore, the proposed DPBHA improved the overall average throughput by 6.15%.

### 5.4. End-To-End Delay

The end-to-end delay describes the time between when the packet is generated at the source node to when the packet is received by the destination node. It is the average time needed for the data packets to be transmitted from the source node to the destination node, as given in Equation (19).

(19) $$E2E\;\mathrm{Delay}=\frac{\sum_{i=1}^{n}\left(\mathrm{Received}\;\mathrm{Packet}\;\mathrm{Timer}-\mathrm{Sent}\;\mathrm{Packet}\;\mathrm{Timer}\right)*1000\left(\mathrm{ms}\right)}{\mathrm{Total}\;\mathrm{Number}\;\mathrm{of}\;\mathrm{Packets}\;\mathrm{Delivered}\;\mathrm{Successfully}}$$

**Figure 13:**
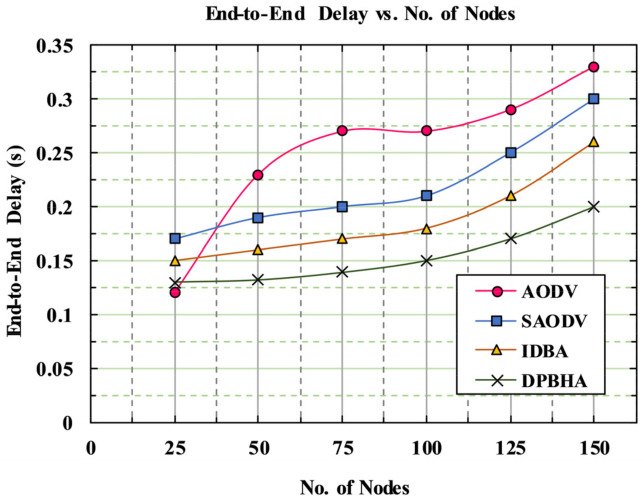
Graphical representation of end-to-end delay.

Figure 13 plots the performance metric of E2E delay (in seconds) for the DPBHA and benchmark schemes. Here, the E2E delay was high when the density of nodes was high. It can be observed that the average E2E delay of the proposed DPBHA was lower than the other schemes. A high PDR leads to a lower E2E delay and optimal throughput due to a large number of packets being delivered to the destination node with less amount of time. The classic AODV shows a significant hike in E2E delay when the number of nodes increased from 25 to 150. The average E2E delay of the conventional AODV was 30.93%; this was because of the presence of more malicious nodes and packet collision events in the network. When the target destination was not reached, a new route discovery process needed to be initiated. Using a combination of the dynamic threshold value and a forged RREQ mechanism, the speed of data transmission increased and the delay decreases in the proposed DPBHA, as shown in Figure 13. This was because the DPBHA quickly detected the malicious nodes from the network and selected the best and most secure route for data transmission. The average E2E delay of the proposed DPBHA was recorded as 18.86%, which is the lowest among all the schemes. Similarly, the average E2E delays of the SADOV and IDBA were recorded as 27.04% and 23.15%, respectively. Hence, the proposed DPBHA reduced the overall average E2E delay by 6.13%.

### 5.5. Packet Loss Rate (PLR)

The packet loss rate (PLR) is the difference between the total number of data packets sent by the source node and the total number of data packets successfully received by the destination node, as given in Equation (20). Usually, packets are lost by malicious nodes or due to increased congestion in the network.

(20) PLR=∑Number of packets sent−∑Number of packets received 

**Figure 14:**
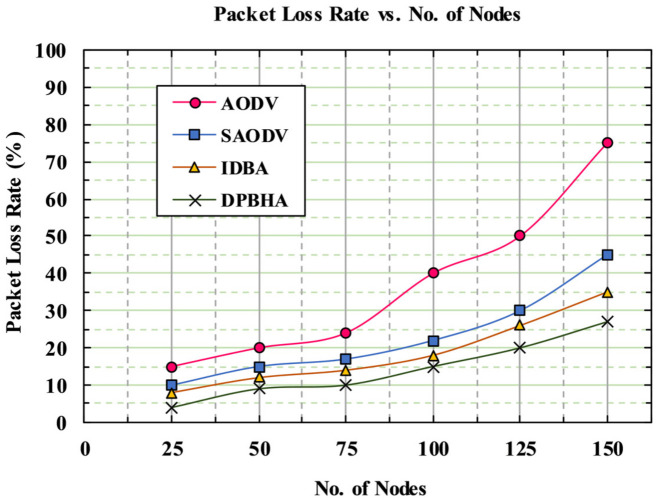
Graphical representation of packet loss rate.

Figure 14 illustrates the simulation results, indicating the PLR in terms of percentage on the *y*-axis and the number of nodes on the *x*-axis. It can be observed that as the number of nodes increased, the PLR increased due to the presence of more malicious nodes and packet collision occurrences in the network. When a malicious node performs a packet-dropping attack, it badly affects the PLR. The classic AODV severely suffered from the presence of a BHA, where an average of 37.33% of packets were lost due to a lack of security mechanisms. It was further observed that the average PLRs for SAODV and IDBA were recorded as 24.77% and 20.14%, respectively. These schemes achieved a good level of performance regarding the PLR because both of them employed some security mechanisms that detect a BHA instantly. Similarly, the proposed DPBHA first identifies the malicious node with the help of a dynamic threshold value and then makes confirms it as BHA by broadcasting a forged RREQ. The PLR for the proposed DPBHA was recorded as 15.15% due to the instant elimination of BHAs. Thus, the proposed DPBHA reduced the overall average PLR by 9.84%.

### 5.6. Confusion Matrix

Intrusion detection systems (IDSs) are usually evaluated based on the following measures of confusion matrix shown in Table 6. The columns of the table represent instances in the predicted class. Similarly, the rows of the table represent instances in the actual class.

#### 5.6.1. True Positive Rate (TPR)

When the model correctly identifies and detects an attacker in a network, it is said to be true positive. The sensitivity or detection ratio is another name for the TPR (DR). It is calculated as the ratio between the predicted attacks and the total number of attacks. Mathematically, the TPR can be calculated using Equation (21).

(21) $$\mathrm{TPR}=\frac{\mathrm{TP}}{\mathrm{TP}+\mathrm{FN}}$$

#### 5.6.2. False Positive Rate (FPR)

When the model misidentifies a legitimate node as an attacker, it is said to be a false positive. FPR is calculated as the ratio of the total number of normal instances that are wrongly classified as an attacker to the overall number of normal instances. Mathematically, the FPR can be expressed using Equation (22).

(22) $$\mathrm{FPR}=\frac{\mathrm{FP}}{\mathrm{FP}+\mathrm{TN}}$$

#### 5.6.3. False Negative Rate (FNR)

A false negative occurs when there are attacker nodes that are incorrectly classified as legitimate or normal nodes. It means that an anomaly is not being detected by the model and is labeled as normal. Mathematically, the FNR can be calculated using Equation (23).

(23) $$\mathrm{FNR}=\frac{\mathrm{FN}}{\mathrm{FN}+\mathrm{TP}}$$

#### 5.6.4. True Negative Rate (TNR)

A true negative occurs when there is no attacker node and the model identifies it as a normal node. It means that the scheme successfully labels legitimate nodes as normal nodes. Mathematically, the TNR can be expressed using Equation (24).

(24) $$\mathrm{TNR}=\frac{\mathrm{TN}}{\mathrm{TN}+\mathrm{FP}}\;$$

#### 5.6.5. Detection Rate

The detection ratio is an important metric to examine the accuracy of a model when identifying and detecting the malicious nodes in a network. Table 7 illustrates the statistical analysis of the detection ratio of the proposed DPBHA and its comparison to the benchmark schemes with the various number of normal and malicious nodes.

**Figure 15:**
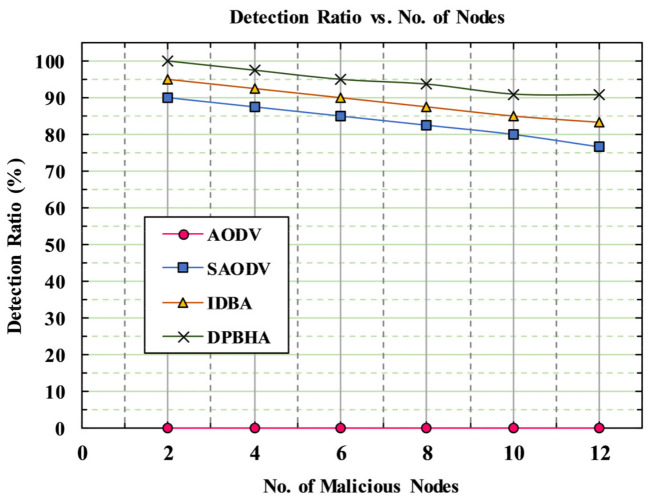
Graphical representation of detection rate.

Figure 15 depicts the simulation results of the detection ratio of the proposed DPBHA and its comparison to the benchmark schemes. The results showed that the average detection ratio of the proposed DPBHA was reported as 94.66%, which was the highest detection rate across all schemes. The main reason for the highest detection rate was the fact that the proposed DPBHA first checks each RREP’s sequence number with the calculated dynamic threshold value. If the received RREP’s sequence number is higher than the threshold value, then that node is detected as a suspicious node with a 50% probability. Further, in the next phase, the suspected malicious node is 100% confirmed that it is a black hole node if it replies to the forged RREQ. This means that the proposed DPBHA can detect and prevent the malicious node instantly and accurately by performing the two-stage approach. As soon as the number of legitimate and malicious nodes increased in the network, the chances of malicious node detection decreased due to an increase in congestion and packet collision occurrences. However, the proposed DPBHA could detect and prevent the BHA more accurately and rapidly than other benchmark schemes. The classic AODV was designed with no security mechanism; therefore, its detection rate was recorded as 0.0%, as shown in Figure 15. The average detection rates for SAODV and IDBA were recorded as 83.6% and 88.88%, respectively. Figure 15 reveals that the proposed DPBHA’s detection ratio was high for a majority of points, with an average of 94.66%.

#### 5.6.6. Accuracy Rate

The accuracy metric measures how accurate the model is in detecting malicious or normal node behavior. It is defined as the percentage of all those correctly predicted instances to the overall instances calculated using Equation (25). In order to maximize the performance of a model, FPR and FNR must be minimized, while TPR and TNR must be maximized.

(25) $$\mathrm{Accuracy}=\frac{\mathrm{TP}+\mathrm{TN}}{\mathrm{TP}+\mathrm{TN}+\mathrm{FP}+\mathrm{FN}}$$

Table 8 demonstrates one of the experiments of the proposed DPBHA performed with a total number of 75 nodes comprising 69 normal and 6 malicious nodes. After performing the simulation, the model successfully detected five out of six targeted malicious nodes, giving an 83.3% sensitivity. In Table 8, the positive predictive value (PPV) means the probability that the model successfully detected the true attacker nodes and is calculated using $\mathrm{PPV}=\mathrm{TP}/\left(\mathrm{TP}+\mathrm{FP}\right)\times 100=5/\left(5+0\right)\times 100=100\%.$ Similarly, the negative predictive value (NPV) means the probability that the model correctly identifies a negative test as a non-attacker node. Mathematically, the NPV is represented by$\;\mathrm{NPV}=\mathrm{TN}/\left(\mathrm{TN}+\mathrm{FN}\right)\times 100=69/\left(69+1\right)\times 100=98.5\%$. Finally, the accuracy rate for the proposed DPBHA was calculated as 98.6%, which is a high accuracy rate for any given model.

## 6. Conclusions and Future Work

Safety and security are the major concerns in VANET applications. Many road applications, such as traffic reports and accident notifications, can strongly support safety requirements. However, VANETs are vulnerable to a variety of security threats and attacks because of their highly dynamic, decentralized nature and protocol design concerns. As a result, VANET applications and services are jeopardized. There is the possibility that VANET applications will have certain security requirements. However, life and safety-critical messages must be sent from V2V in a secure and timely way. Because vehicles exchange messages at fast speeds over an open wireless medium, ensuring the security of these messages is critical. The security aspect of VANETs was the focus of this research work. To protect and improve the overall performance of VANETs, an innovative and effective solution was proposed called DPBHA, which could detect and prevent black hole security attacks in the AODV routing protocol. The solution was based on calculating a dynamic threshold value and generating a forged RREQ packet. The proposed DPBHA was implemented and evaluated in the NS-2 simulator, and its performance and efficacy were compared to the benchmark schemes. In conclusion, we showed that the proposed DPBHA outperformed the benchmark schemes in terms of improved PDR by 3.0%, increased throughput by 6.15%, reduced routing overhead by 3.69%, decreased E2E delay by 6.13%, reduced PLR by 9.84%, and achieved a maximum detection rate of 94.66%.

Future research includes detecting and preventing gray hole security attacks, which are considered to be some of the severe attacks on VANETs. Similarly, more efforts will be made in the future to explore state-of-the-art advancements in the field and address various security issues associated with vehicular networks.

## Figures and Tables

**Table 1:** Summarized literature review.

Author (s) and Citation	Solutions/Schemes	Strengths	Performance Metrics	Limitations
Hortelano et al. [28]	Watchdog-based IDS	Easy to implement and applicable in any routing protocol; detects selfish and greedy nodes efficiently	False positive and false negative	The technique fails when two malicious nodes work together; a high false detection rate in a short time; generates a huge routing overhead and end-to-end (E2E) delay
Daeinabi et al. [29]	Detecting malicious vehicle (DMV)	Detect any kind of malicious node with high promptness	PDR and packets dropped	High jitter and high E2E delay; low throughput
Kadam et al. [30]	Detection and prevention of malicious vehicles (D&PMV)	Provides lower jitter and higher throughput compared to DMV method	Packets dropped, E2E delay, throughput, and jitter	Requires more time for processing; results in high E2E delay
Dhaka et al. [31]	Based on new control packets: Cseq and Rseq	Provides higher PDR and is applicable in other reactive routing protocols	PDR and E2E delay	Huge routing overhead due to use of additional control packets
Jahan and Suman [32]	Acknowledgment-based model	The model is capable of detecting any kind of malicious node	Packets dropped, throughput, packets received, and PDR	Heavy routing overhead and E2E delay; low throughput and PDR
Li et al. [33]	Attack-resistant trust (ART) management scheme based on evaluating trustworthiness	Accurately evaluates the trustworthiness of data and nodes in VANETs; capable of detecting various DoS attacks	Precision, recall, and communication overhead	High processing overhead when the number of malicious nodes increases; cannot detect a smart BHA
Purohit et al. [34]	Secure vehicular on-demand routing (SVODR)	The modified AODV can mitigate the impact of BHAs in VANETs	PDR, throughput, normalized routing load (NRL), E2E delay, and average path length	It cannot be employed with other protocols; using extra fields for cryptographic functions leads to a heavy routing overhead and E2E delay
Tyagi et al. [35]	Enhanced secure AODV (ES-AODV) based on asymmetric public-key cryptography	The algorithm is simple, fast, and has a lower storage cost	Packets dropped, packet collision, E2E delay, throughput, routing overhead, and PDR	Provides security against external attacks but internal attacks may inflict havoc on the network
Zardari et al. [36]	Dual-attack detection for a BHA and GHA (DDBG)	Provides a fast propagation rate of data and only trustworthy nodes can interact across the network	Detection rate, PDR, throughput, routing overhead, and E2E delay	Generates a huge routing overhead, which affects the throughput and PDR
Cherkaoui et al. [4]	Use of variable control chart to detect BHA	Easy to implement and does not need any modification in the routing protocols	Throughput and E2E delay	High processing overhead and may not apply in the VANET’s environment
Hassan et al. [20]	Intelligent detection of a black hole attack (IDBA)	Capable of detecting a BHA and the results revealed better performance compared to benchmark schemes	PDR, throughput E2E delay, packet loss ratio, and routing overhead	Generates four thresholds, which causes a high processing and routing overhead
Kumar et al. [10]	Secure AODV	Capable of detecting malicious nodes in VANETs	PDR, throughput, and E2E delay	High routing overhead and E2E delay, resulting in a decreased throughput and PDR
Proposed DPBHA	Use of dynamic threshold value and forged RREQ packet	Efficiently detects and prevents a BHA in terms of reduced routing overhead and E2E delay, increased throughput, and PDR; eliminates the false positive and false negative rates with 98% accuracy; no additional hardware and IDS/DPS nodes are required	PDR, throughput, E2E delay, packet loss ratio, routing overhead, and detection ratio	The proposed DPBHA addresses BHA only and it is incapable of addressing other DoS attacks, such as cooperative BHA and GHA, which will be addressed in future research work

**Table 2:** Notations and their descriptions.

Symbol	Description
N	Node: vehicle or RSU
S	Source node
D	Destination node
E	Edge
T	Timer
V	Vehicle
$N_{N}$	Neighboring node
$N_{\mathrm{HN}}$	Next-hop node
$R_{T\;}$	Routing table
$V_{N_{N}}$	Speed of neighboring node
ID	Identity of a node
G	Gray list
B	Black list
RREQ	Route request
RREP	Route reply
$T_{R\;}$	Transmission range
σ	Standard deviation
$F_{V}\left(V\right)$	Probability density function of a vehicle’s velocity
$D_{\mathrm{SN}}$	${\mathrm{Destination}}_{sequence\;number}$
µ	Mean value
P	The density of vehicles
Λ	Threshold value (sequence numbers)
I and j	Variables i and j range from 1, 2, 3, …, n

**Table 3:** RAT with the normal and malicious nodes’ RREPs.

$N_{ID}\;\left(RREP_{i}\right)$	$D_{SN}$	Hop Count
1	400	1
D	95	1
8	80	4
5	75	2

**Table 4:** The format of a forged RREQ packet.

Packet Type	Flags	Reserved	Hop Count
RREQ (Broadcast) ID			
(Non-existing Destination IP Address)			
Destination Sequence Number			
Originator IP Address			

**Table 5:** Simulation parameters.

S. No.	Parameters	Values
1.	Simulation tool	NS-2.35
2.	Simulation area	1000 m × 1000 m
3.	Number of nodes	25, 50, 75, 100, 125, 150
4.	Simulation time	900 s
5.	Vehicle mobility	1 km/h–100 km/h
6.	Routing protocols	AODV
7.	Standard protocol	802.11p
8.	Black hole nodes	2, 4, 6, 8, 10, 12
9.	Transport protocol	UDP
10.	Packet size (bytes)	512 b/s
11.	Type of traffic	CBR (1 Mbps)
12.	Antenna	Omni-directional

**Table 6:** Confusion matrix.

	Actual Reality Class		
**Test Result Class**	Class	Attack	Normal
	Attack	True positive (TP)	False positive (FP)
	Normal	False negative (FN)	True negative (TN)

**Table 7:** Detection ratio evaluation values.

No. of Nodes	Malicious Nodes	TPR of AODV	TPR of SAODV	TPR of IDBA	TPR of DPBHA
25	2	00.0%	90.0%	95.0%	100%
50	4	00.0%	87.5%	92.5%	97.5%
75	6	00.0%	85.0%	90.0%	95.0%
100	8	00.0%	82.5%	87.5%	93.7%
125	10	00.0%	80.0%	85.0%	91.0%
150	12	00.0%	76.6%	83.3%	90.8%

**Table 8:** Example of an accuracy rate calculation.

Total No. of Nodes = 75		Real Class		Predictive Value
		Attacker = 06	Normal = 69	
**Test Results Class**	Attacker = 5	True Positive = 5	False Positive = 0	Positive Predictive Value (5/5) = 100%
	Normal = 70	False Negative = 5	True Negative = 69	Negative Predictive Value (69/70) = 98.5%
**Results**		Sensitivity (5/6) = 83.3%	Specificity (69/69) = 100%	$\mathrm{Accuracy}=\frac{5+69}{5+69+0+1}\times 100$ =98.6%

## Data Availability

The simulation files/data used to support the findings of this study are available from the corresponding author upon request.
